# Influence of Shortness of the Umbilical Cord on Labor

**Published:** 1845-05

**Authors:** 


*Influence of shortness of the Umbilical Cord on Labor.*---MM. Capuron and Danyau read to the French Academy of Medicine a report on a memoir presented by M. Hirtz, of Strasbourg, on this subject. M. Hirtz believes, along with many old and modern accoucheurs, that shortness of the cord, either natural or secondary, (when it is rolled around the neck of the fœtus,) is a cause of difficult labor, and warrants, indeed necessitates, the use of the forceps, as soon as possible after it has been recognized. It may, he says, be recognized by the following symptoms: The alternating descent and ascent of the head in the vagina, occasioned by the uterus dragging up the fœtus, when it ascends after being pressed downwards by the contraction of the abdominal muscles; local pain experienced in that part of the uterus to which the placenta is attached, and caused by the dragging of the cord. The reporters, in contradiction to M. Hirtz, do not appear to admit that shortness of the cord is an impediment to labor, and deny entirely the value of the symptoms given by M. Hirtz, which may be said to sum up those of former writers. They think that the ascent of the fœtal head in the interval of uterine contraction is the result of the elasticity of the bones of the head, and of the parts which cover the parietes of the maternal pelvis. Moreover, the uterus, being in a state of relaxation, cannot exercise any traction on the fœtus. Even in cases in which the head of the fœtus appears to traverse nearly the entire length of the vagina, in its alternate descent and ascent, they think that the circumstance is to be attributed merely to the above causes, viz: the elasticity of the head and parietes of the pelvis. As to the local uterine pain, it is often observed even in the most natural labors, and is, consequently, of little or no value. They do not find, either, that the adoption of these views has influenced the practice of M. Hirtz. In the cases which he narrates, the patients being primiparous, the head remaining much longer than usual in the cavity of the pelvis, and there being local uterine pain, M. Hirtz applied the forceps. Finding the cord twisted round the neck of the fœtus, he concluded that this was the cause of the tardy delivery. Such an opinion, although legitimate, is not conclusive, as it is merely founded on the coincidence of a tedious labor in a primiparous woman, with twisting of the cord round the fœtal neck. The application of the forceps was indicated in such a case without reference to shortness of the cord.---*Lancet*, Jan. 4, '45, in *Am. Jour. of Med. Sci.*

